# Rational speech comprehension: Interaction between predictability, acoustic signal, and noise

**DOI:** 10.3389/fpsyg.2022.914239

**Published:** 2022-12-16

**Authors:** Marjolein Van Os, Jutta Kray, Vera Demberg

**Affiliations:** ^1^Department of Language Science and Technology, Saarland University, Saarbrücken, Germany; ^2^Department of Psychology, Saarland University, Saarbrücken, Germany; ^3^Department of Computer Science, Saarland University, Saarbrücken, Germany

**Keywords:** speech comprehension, background noise, mishearing, predictive context, rational processing, noisy channel

## Abstract

**Introduction:**

During speech comprehension, multiple sources of information are available to listeners, which are combined to guide the recognition process. Models of speech comprehension posit that when the acoustic speech signal is obscured, listeners rely more on information from other sources. However, these models take into account only word frequency information and local contexts (surrounding syllables), but not sentence-level information. To date, empirical studies investigating predictability effects in noise did not carefully control the tested speech sounds, while the literature investigating the effect of background noise on the recognition of speech sounds does not manipulate sentence predictability. Additionally, studies on the effect of background noise show conflicting results regarding which noise type affects speech comprehension most. We address this in the present experiment.

**Methods:**

We investigate how listeners combine information from different sources when listening to sentences embedded in background noise. We manipulate top-down predictability, type of noise, and characteristics of the acoustic signal, thus creating conditions which differ in the extent to which a specific speech sound is masked in a way that is grounded in prior work on the confusability of speech sounds in noise. Participants complete an online word recognition experiment.

**Results and discussion:**

The results show that participants rely more on the provided sentence context when the acoustic signal is harder to process. This is the case even when interactions of the background noise and speech sounds lead to small differences in intelligibility. Listeners probabilistically combine top-down predictions based on context with noisy bottom-up information from the acoustic signal, leading to a trade-off between the different types of information that is dependent on the combination of a specific type of background noise and speech sound.

## 1. Introduction

When we are trying to understand other people’s speech, there are at least two sources of information available. First, there is the bottom-up sensory information in the form of the acoustic speech signal. This can be masked by background noise, hindering speech recognition. Second, there is top-down information from the language system, for example word frequencies or possible grammatical constructions. Models of speech recognition posit that different sources of information are combined (e.g., FMLP, Oden and Massaro, 1978; NAM, Luce and Pisoni, 1998; Shortlist B, Norris and McQueen, 2008; see also Samuel, 1981), often based on Bayesian principles. Also the sentence context is a form of top-down information that can guide predictions during listening (Boothroyd and Nittrouer, 1988; Nittrouer and Boothroyd, 1990; Altmann and Kamide, 1999; Pickering and Gambi, 2018).

Many studies have investigated how these different factors, predictability, background noise, and the interaction with certain acoustic features, affect speech comprehension. However, the three have not been combined in a single study: Previous studies that investigated predictability effects in noise did not carefully control the types of sounds and how they are affected by noise (Kalikow et al., 1977; Boothroyd and Nittrouer, 1988; Hutchinson, 1989; Pichora-Fuller et al., 1995; Sommers and Danielson, 1999; Dubno et al., 2000), while the literature on effects of background noise on speech sounds does not specifically manipulate predictability effects in sentence comprehension (Pickett, 1957; Gordon-Salant, 1985; Phatak et al., 2008; Cooke, 2009; Alwan et al., 2011). Additionally, results on the effect of background noise are inconclusive regarding which type of noise affects comprehension most severely (Horii et al., 1971; Danhauer and Leppler, 1979; Gordon-Salant, 1985; Nittrouer et al., 2003; Taitelbaum-Swead and Fostick, 2016). The present study aims to fill this gap. Our stimuli contain two different types of noise, multi-speaker babble noise and white noise, as well as silence, and specific sound contrasts within the target words. These sound contrasts are voiced and unvoiced plosives differing in the place of articulation, voiceless fricatives and affricates differing in place of articulation, or vowels that are either tense or lax. We are particularly interested in the interaction of noise type and sound contrast on recognition in context. These conditions lead to small changes in intelligibility depending on the exact combination of noise and speech sounds, which have previously primarily been investigated in isolated syllables (e.g., Miller and Nicely, 1955; Wang and Bilger, 1973; Gordon-Salant, 1985; Benkí, 2003). Our research also has potential for practical applicability to better intelligibility of machine-generated speech: If models of top-down predictions and interference between the bottom-up signal and the environmental noise are available, the formulation of a message has optimal intelligibility in the current listening conditions can be preferably chosen over alternatives which carry higher risk of being misunderstood (Cooke et al., 2014; Chingacham et al., 2021).

### 1.1. Related work

#### 1.1.1. Models of speech perception

Through the years, several models of speech comprehension have been proposed. Many of these models capture the idea that information from several sources (the speech signal, lexical frequency, or context for example) are combined in the recognition process, and use Bayesian principles to simulate a rational listener. For example, the Fuzzy Logical Model of Perception (FLMP; Oden and Massaro, 1978) assumes that speech recognition should be optimal by independently evaluating different sources of information. There is a trade-off in the model where context plays a larger role when the phonetic information is ambiguous. The Neighborhood Activation Model (NAM; Luce and Pisoni, 1998) is based on word frequencies as well as the concept of similarity neighborhoods: a set of words that are phonetically similar to a target word. It also takes into account stimulus word intelligibility as the input of the model is data from an experiment where listeners identified CVC words in background noise. A limitation of this model is that it can only account for isolated monosyllabic words, rather than a continuous speech stream. Shortlist B (Norris and McQueen, 2008) is a model that is capable of this. It is a Bayesian model that integrates bottom-up and top-down signals to simulate an optimal listener. Its input is the data of a gating study of CV and VC syllables. This data set provides perceptual confusions, like in the NAM, but in quiet rather than background noise, and also provides time-course information of the confusions. According to these latter two models, optimal word recognition depends on bottom-up evidence (from the acoustic signal) and prior lexical probabilities (based on frequencies). Shortlist B additionally considers contextual information to affect the priors. In particular, both contextual information and word frequency will influence recognition when the perceptual evidence is poor and decrease as the perceptual evidence improves.

However, these models are generally based on empirical data from studies investigating the perception of mono-syllabic words rather than sentences or larger contexts. Additionally, they primarily focus on explaining effects of frequency and small local contexts consisting of surrounding syllables. In our experiment, we will make use of sentences with a predictable or unpredictable context embedded in background noise, to test how listeners combine different sources of information during speech comprehension. We do not manipulate word frequency, but word predictability, and use an entire sentence to set up the participants’ expectations. Still, our predictions are based on similar Bayesian principles as have been used in the models of speech perception.

#### 1.1.2. Predictability

Predictability affects language comprehension, so that generally the more predictable a word is, the easier it is to process and integrate. This effect has been shown in different aspects of language processing. Responses on a cloze task are faster in sentences with a high predictability as measured by cloze value (Nebes et al., 1986; Staub et al., 2015). Words are read faster or even skipped when they have a high predictability rather than a lower predictability (Ehrlich and Rayner, 1981; Kliegl et al., 2006; Smith and Levy, 2013). Conversely, low predictability and violations of plausibility lead to processing difficulties as shown by longer reading times (Rayner et al., 2004; Staub et al., 2007; Warren and McConnell, 2007). Inaccurate predictions cause recognition rates to be worse the farther along in a sentence the listener gets, as the wrong predictions lead to misunderstandings (Marrufo-Pérez et al., 2019). Also neural responses are affected by predictability, with both the N400 and P600 components being modulated by context (Kutas and Hillyard, 1984; DeLong et al., 2005; Van Berkum et al., 2005; Van Petten and Luka, 2012; Aurnhammer et al., 2021). Taken together, these results suggest that a word’s context can facilitate comprehension of that word if it is predictable, while in cases of a low predictability context comprehension is hindered.

This facilitatory effect of a predictable context is present also in background noise, leading to improved recognition (Kalikow et al., 1977; Boothroyd and Nittrouer, 1988; Hutchinson, 1989; Pichora-Fuller et al., 1995; Sommers and Danielson, 1999; Dubno et al., 2000). Kalikow et al. (1977) set out to construct a controlled test of speech intelligibility in noise (SPIN) where they varied the predictability of the target words’ context. The sentences were either highly predictable or had a neutral carrier phrase to have low predictability. Normal hearing subjects were presented with the sentences embedded in multi-speaker babble noise at different SNRs. They performed differently on the items with high versus low predictability, with higher accuracy for the predictable items. Similar results were found by Boothroyd and Nittrouer (1988) testing CVC syllables (existing words vs. nonsense words) and four-word sentences (high predictable, low predictable, and random sequences of words). By relying on context, the adverse effect of noise can even be overcome (Wingfield et al., 1995, 2005). However, it can also lead to mishearings when the predictions are wrong or the context is misleading (Rogers et al., 2012; Sommers et al., 2015; Failes et al., 2020). Connine (1987), Spencer and Halwes (1981), and Miller et al. (1984) investigated specifically the effect of context on the interpretation of minimal pairs with a spectrum of ambiguous and unambiguous voice onset time (for example on a continuum between DENT and TENT). The results showed the sentence context affected the participants’ response in that they followed the bias provided by the sentence.

The present study likewise investigates the interaction of acoustic and contextual information, but rather than having ambiguous sounds, we add noise to the signal to investigate listener’s behavior in adverse conditions.

#### 1.1.3. Background noise

Studies investigating speech comprehension in noise have shown that human listeners are quite robust against sudden noise overlapping with the speech signal. Phonemic restoration refers to the phenomenon where listeners believe they heard the missing sounds in cases when a cough, a tone, or burst of noise replaces a phoneme completely (Warren, 1970; Sasaki, 1980; Kashino, 2006). The effect is not observed when the phoneme is replaced with silence, or the replacing noise is fainter than the speech signal. Listeners seem to make use of contextual information during the phonemic restoration process: in ambiguous cases, listeners report hearing the sound that completes the word to fit the context it is in rather than the actually pronounced similar sounding word (*sandwagon* was presented instead of *bandwagon*; Warren, 1970; Warren and Sherman, 1974). Additionally, Bashford et al. (1992) found no effect of phonemic restoration on the intelligibility of tested word lists, and only limited benefit in low predictability sentences, compared to a larger benefit in high predictability sentences. To avoid phonemic restoration effects, we present our participants with sentences completely embedded in background noise, and not just the target sound. This way, the noise is part of the signal rather than a short tone that is edited out in the processing of the speech.

Background noise has an adverse effect on speech comprehension due to various types of masking. In the present article, we will focus on energetic masking, where both the speech signal and the masking noise have energy in the same frequencies at the same time (Brungart, 2001). In this way, the noise masks the acoustic cues listeners use for sound identification. Various types of noise affect the speech signal in distinctive ways. This occurs due to spectral differences (Gordon-Salant, 1985). Babble noise continuously varies in amplitude, while white noise is stationary (Weber and Smits, 2003). Multi-speaker babble noise approximates the average long-term spectrum of the speech of a single speaker (Simpson and Cooke, 2005; Garcia Lecumberri et al., 2010), whereas white noise has a flat spectral density with the same amplitude throughout the audible frequency range (20–20,000 Hertz). Both types of noise will be tested in the present study.

Previous studies have used both babble noise and white noise to test speech intelligibility in background noise. Gordon-Salant (1985) used multi-speaker babble noise, testing 57 CV sequences, and comparing results to previous similar studies using white noise maskers (e.g., Soli and Arabie, 1979). The results showed that the interference effects of both noise types differ from each other, particularly in higher levels of noise. Taitelbaum-Swead and Fostick (2016) found lower accuracy for white noise than babble noise and speech-shaped noise (frequencies between 0.5 and 2 kHz with constant amplitude) when testing the intelligibility of both meaningful and nonsense words. Nittrouer et al. (2003) found a recognition advantage in speech-shaped noise compared to white noise with phonetically balanced monosyllabic words for adults, while children show impaired recognition. Other studies found opposite effects of noise type, with worse performance in speech-shaped noise maskers compared to white noise maskers (Horii et al., 1971), which Carhart et al. (1975) attributed to difficulty to separate target speech from a speech competitor. Danhauer and Leppler (1979) found similar masking results for white noise and cocktail party noise. These varying findings with regards to noise type effects suggests that the type of task and exact stimuli used, as well as the tested population and the level of the noise signal relative to the speech affect these effects.

#### 1.1.4. Speech sound contrasts

We are interested in the interaction of background noise and speech sound contrasts. The effect of the different types of noise might also be modulated by the type of speech sounds present in the stimuli, as there are different masking effects depending on the exact acoustic signal. In our stimuli, we used pairs of fricatives and affricates, pairs of plosives, and pairs of vowels. These sounds differ in their acoustic characteristics, affecting their recognition. Plosives consist of a closure of some part of the vocal tract, followed by a short burst of energy (Ladefoged and Maddieson, 1996). This short burst is easily lost in general background noise, impeding recognition. In this study, the plosive pairs will differ only in place of articulation, voicing is kept the same across pairs. Spectral frequency information and formant transitions have been found to be particularly important for identifying the place of articulation in plosives (Liberman et al., 1954; Edwards, 1981; Alwan et al., 2011). This information can easily be lost in noise, making correct recognition difficult (Gordon-Salant, 1985; Weber and Smits, 2003; Phatak et al., 2008). We also test pairs of fricatives and affricates. These sounds are made by forcing air through a narrow channel in the vocal tract, causing a turbulant airflow. They have a greater constancy of shape in different phonetic contexts compared to plosives (Ladefoged and Maddieson, 1996). The turbulent noise in fricatives is irregular and random, like in white noise, albeit less flat (Johnson, 2003). Important cues for the recognition of place of articulation in fricatives are, like for plosives, the relative spectral amplitudes, as well as other characteristics like duration (You, 1979; Alwan et al., 2011). Vowels are defined by having no major constrictions in the vocal tract (Ladefoged and Maddieson, 1996). The exact position of the tongue in terms of height and backness determines the vowel sound, also known as the first and second formants. While different features are being distinguished to describe vowels, for the present study the distinction between tense and lax is most important. The difference between a tense and a lax vowel of a pair is in their height and backness. Generally, lax vowels tend to be more centralized than their tense counterparts, while tense vowels are more peripheral in the vowel space. Another difference lies in the force input of articulation (Hoole and Mooshammer, 2002).

The recognition of speech sounds is impaired by background noise, as the competing noise obscures the cues that are needed for recognition. Most studies have investigated the recognition of consonants, usually in Consonant-Vowel (CV) or Vowel-Consonant (VC) syllables with fixed vowel contexts. Gordon-Salant (1985) asked normal-hearing participants to identify CV syllables embedded in multi-speaker babble noise. She found that in higher levels of multi-talker babble noise, fricatives are identified more accurately than plosives. On the other hand, studies using a similar design found that in severe levels of white noise the recognition of fricatives is reduced (Miller and Nicely, 1955; Wang and Bilger, 1973; Phatak et al., 2008). Using a multi-speaker babble noise, Weber and Smits (2003) tested all possible CV and VC syllables in English in order to test recognition of vowels and consonants both in syllable-initial and syllable-final position. They found that vowels were recognized better than consonants in general, while plosives and fricatives led to the lowest number of correct responses. Mistakes that were made in the recognition of plosives were mostly errors regarding the place of articulation. Fricatives had a larger variety of errors, which were in manner, place, and voicing.

Phatak et al. (2008) compared the effect of white noise as a masker to a previous study that used speech weighted noise (Phatak and Allen, 2007). They found that the tested consonants (English voiced and voiceless plosives, fricatives, and nasals) were recognized more poorly in white noise compared to speech weighted noise, but that the recognition of sibilant fricatives in particular is most reduced in white noise. A study by Woods et al. (2010a, see also 2010b) investigated the recognition of CVC syllables in speech-spectrum noise, measuring not only hit rate, but also two metrics from signal detection theory, namely *d’* and *beta*. d’ is a measure of sensitivity that is based on hit rates and false alarm rates, while beta is a measure of response bias, reflecting the trade-off between detecting the signal when present and not reporting it when absent. Their results were very similar to those of Miller and Nicely (1955) and Phatak and Allen (2007), showing that consonants are affected differently by noise. Confusion clusters showed large amounts of confusion within consonants of the same type (voiced or unvoiced plosives cluster together, as do fricatives), and place confusions were common. Alwan et al. (2011) compared paired plosives and fricatives that differed only in their place of articulation. In a two-alternative forced choice task when listening in white noise, they found that fricatives were more robust than plosives.

When it comes to the recognition of vowels in background noise, Parikh and Loizou (2005) tested the recognition of vowels and stops. They found that in higher levels of speech-shaped noise, the second formant is heavily masked, while the first formant was reliably detected in noise. Pickett (1957) investigated the recognition of vowels in different types of background noise, and found that the confusions participants made change when the noise changes, depending on how much the frequencies of noise and speech overlap and whether one of the formants is still intelligible. A study by Benkí (2003) tested CVC syllables and investigated both the recognition of onset and coda consonant, as well as the vowel. Results were in line with previous studies (Miller and Nicely, 1955; Pickett, 1957; Wang and Bilger, 1973), showing for example that word-initial consonants are more robust to noise masking than word-final consonants, that place of articulation is easily masked.

In the present study, we will investigate the effect of both multi-speaker babble noise and white noise on the recognition of pairs of speech sounds, with a design that allows us to compare both the effect of either noise on the speech sound category, and the categories to each other. We expect that recognition performance will be poorer in noise than quiet, particularly for the plosives. Fricatives will be harder to recognize correctly in white noise than in babble noise, with the opposite effect for vowels.

### 1.2. Research goals and hypotheses

The aim of the present study is to investigate how listeners combine different types of information when listening to sentences in background noise, varying the amount of background noise through the interaction of noise type and speech sound. We fill the gap in the empirical literature where these factors have often been investigated separately, but not combined in one experiment. We examined mishearings that occur when listening in background noise, depending on predictability of the context and certain sound characteristics of the target word. We carefully controlled the target word so that it formed a minimal pair that differed in a medial sound contrast, controlling the specific pairs of sound contrasts. In this experiment, we presented participants with a written sentence context on the screen that can be used to guide prediction while listening. In the high predictability condition, these predictions would lead to the correct response, but in the low predictability condition relying on the context gives an incorrect response.

We expected to find a main effect of noise: overall, there will be a lower number of correct responses in noise compared to quiet. This result acts as a control condition: finding fewer correct responses in quiet than noise would point to a problem in the experimental design. We additionally investigate whether there is a difference between the two noise types we use, babble noise and white noise. Overall, studies have found conflicting results when comparing white noise and babble noise (e.g., Horii et al., 1971; Gordon-Salant, 1985; Taitelbaum-Swead and Fostick, 2016). Because of these conflicting results, we hypothesize that this effect of noise type most likely depends on other factors, such as the exact task, population, and most importantly the characteristics of the stimuli, such as the presence of predictive context and the occurring phonemes (details of this interaction of noise type and speech sound contrast will be discussed below).

We expected an interaction of noise and predictability based on the semantic context. According to models of speech perception (e.g., Oden and Massaro, 1978; Luce and Pisoni, 1998; Norris and McQueen, 2008), participants should rely more on the sentence context rather than the acoustic signal, when listening in noise. They use the information from the context to compensate for the processing difficulties of the speech signal in background noise (Wingfield et al., 1995, 2005; Gibson et al., 2013; Failes et al., 2020). In the low predictability sentences, this will lead to incorrect responses, as the context is misleading by predicting a different word than the target. As such, we expected that the low predictability condition leads to more mishearing than the high predictability condition. In contrast, in the high predictability condition, the target word is supported by both the audio signal and the context, which should lead to high accuracy rates independent of the noise condition.

In the current study, we manipulate the amount of noise on the signal by using different types of background noise and speech sound contrasts. The interaction of noise type (babble and white noise) and sound contrast (plosives, vowels, and fricatives) should lead to different levels of signal masking, defined by the amount of interference the background noise has on the speech sound. With their burst, plosives have the shortest and least clear signal out of our three tested sound contrasts. Therefore, we expected that plosives will show more mishearing than fricatives and vowels, overall, because the perceived noise is greater. We do not predict any differences in the degree of mishearing in plosives depending on the type of noise, as the signal of the plosive is easily lost in general, but does not overlap in particular with a specific type of noise tested here. We do expect this interaction with noise type for fricatives and vowels. Fricative sounds have their energy at the same frequencies as white noise, and therefore fricatives should be harder to identify correctly in white noise than babble noise. There would be more noise in the form of (energetic) masking in the case of white noise, lowering performance for fricatives (Miller and Nicely, 1955; Phatak et al., 2008). Results for fricatives in white noise might show a low performance with a large amount of mishearing, possibly on the level of the plosives (which are difficult in general). In babble noise, with energy mainly in different frequencies (Simpson and Cooke, 2005; Garcia Lecumberri et al., 2010), recognition of fricatives should not be majorly affected, as the perceived noise is lower. For vowels, we hypothesized that these items generally tend to be more difficult to identify correctly in babble noise, but easier in white noise. In fluctuating babble noise, the particular formant values that determine the vowel might be lost, while in the steady signal of white noise they can be recovered (Pickett, 1957; Benkí, 2003; Weber and Smits, 2003).

In sum, we aimed to investigate how listeners combine different types of information when listening, combining different factors like background noise, characteristics of the speech, and context. We were interested in interactions between noise and predictability on one hand, and noise and sound contrast on the other. We predict that participants’ interpretations will be based more on sentence context in background noise, leading to incorrect responses in the low predictability noise conditions, and that these effects should be modulated by how much the type of noise and the speech sound characteristics overlap in their acoustic signal.

## 2. Materials and methods

### 2.1. Participants

Fifty native speakers of German were recruited for the experiment *via* the recruitment platform Prolific (prolific.co). Data from two participants was excluded due to technical problems. The mean age of the final group of 48 participants was 23 years (age range = 18–30 years), 25 were male. All participants gave informed consent before the experiment, and the study was approved by the Deutsche Gesellschaft für Sprachwissenschaft (DgfS) ethics committee. The experiment lasted approximately 30 min and all participants received €4,75 as compensation for their participation.

### 2.2. Materials

We selected 180 German minimal pairs from the CELEX lexical database (Baayen et al., 1995) with the contrast in the middle of the word (rather than word-initial or word-final). We used three different types of contrasts, namely (1) plosives differing in place of articulation (p/t, p/k, t/k, b/d, b/g, d/g; 62 pairs); (2) vowel contrast pairs with a tense and lax member (i/ɪ, y/ʏ, u/ʊ, ɛ/ɶ, o/ɔ, ɐ/ə; 58 pairs); and (3) voiceless fricative and affricate pairs (f/h, f/ʃ, f/s, f/x, h/ts, pf/ʃ, pf/ts, ʃ/s, s/x, ʃ/ts, s/x, s/ts). This gave a set of 29 pairs. To increase the number of fricative and affricate pairs, we also included minimal pairs that consisted of a (af)fricative and a deletion of the sound (f/ø, ʃ/ø, s/ø, x/ø, ts/ø; together with the fricative pairs *N* = 60). We made sure that all minimal pairs that we selected matched in part of speech and gender in case of nouns, to make construction of the sentences easier. Controlling these factors, however, meant we were not able to control for lexical frequency or neighborhood effects.

We then constructed sentences in which the target word occurred in sentence-final position. There were two levels of predictability. For the high predictability (HP) condition, the target word was predictable from the preceding sentence context and served as a control condition. All stimuli were tested for cloze in an online experiment^1^ with German native speakers so that we had 10 responses for each item. The cloze value was calculated as the number of participants who responded correctly divided by the number of all participants. Items with too low cloze were revised and retested. In these revised versions, we tried to guide participants’ predictions to the target word we had in mind, and changed the items based on their previous responses. We changed or narrowed down the context, or included the alternative candidates in the sentence to get more participants to converge on the target word. We aimed to have high cloze probabilities, but in the final set of 360 high predictability items, 118 still had cloze values under 0.5. The 242 items with cloze values of 0.5 or higher had a mean cloze of 0.75 (SD = 0.17). We relaxed the high cloze requirement for items when even after multiple revisions, there was a high cloze competitor that differed only in the prefix (*laden* vs. *aufladen* for ‘to charge’) or that was too highly frequent and semantically similar to allow us to improve the sentence (*sieden* vs. more frequent *kochen* for ‘to boil’). We included these items even though they had a lower cloze probability than 0.5. The final mean cloze value for all items was 0.59 (SD = 0.27). The items for the low predictability condition (LP) were made by swapping the target words in the HP sentences of a pair, yielding unpredictable and often implausible sentences. For all items, both HP and LP, plausibility ratings were collected using a five-point rating scale ranging from 1 (completely implausible) to 5 (completely plausible). The high predictability items had a mean plausibility rating of 4.60 (SD = 0.65), the low predictability items had a mean rating of 1.69 (SD = 0.90). Example stimuli can be found in Table 1. All items with corresponding cloze values, plausibility ratings, and IPA transcriptions are available on the Open Science Framework^2^. Participants that rated or completed items in the pretests did not participate in the main experiment.

**Table 1:** Example stimuli.

1A	Am Pool im Hotel gab es nur noch eine freie **Liege**.	HP
	*At the pool in the hotel there was only one free* ***lounger*** *left*.	
1B	Nach vier Jahren heiratete Paul seine große **Liebe**.	HP
	*After four years, Paul married his big* ***love***.	
1C	Am Pool im Hotel gab es nur noch eine freie **Liebe**.	LP
	*At the pool in the hotel there was only one free* ***love*** *left*.	
1D	Nach vier Jahren heiratete Paul seine große **Liege**.	LP
	*After four years, Paul married his big* ***lounger***.	

Recordings were made of all 360 high predictability sentences while being read by a female native speaker of German. She was instructed to read slowly and naturally, and to restart when there were any slips of the tongue or hesitations. The LP sentences were constructed *via* cross-splicing of the recordings of the predictable sentences. We did not record the LP sentences to make sure that the intonation and stress patterns would be the same across predictability conditions, and not indicative of the more implausible LP items. The splicing was done using Praat (Boersma and Weenink, 2021, version 6.1.05) and checked by the first author as well as a native German-speaking student assistant to minimize any problems related to the cross-splicing. Taken together, the recordings and cross-splicing resulted in a total of 720 experimental items.

We had three noise conditions, quiet and two types of background noise. The first was a white noise, the second a multi-speaker babble noise where none of the speakers were intelligible (café noise, BBC Sound Effects Library, Crowds: Interior, Dinner-Dance^3^). All items had a Signal-to-Noise ratio (SNR) of −5 dB, meaning the background noise was five dB louder than the sentence-final target word. Because the intensity of a spoken sentence tends to drop toward the end (Vaissière, 1983), it would mean the SNRs were lower for the target word, and thus more difficult, in case the mean sentence intensity was to be used. We calculated the level of background noise separately for all items, both in the HP and LP condition. For each item, the intensity of the target word was measured in Praat (Boersma and Weenink, 2021, version 6.1.05) and the corresponding noise level calculated and automatically mixed in using a Python script. The noise was the same level throughout the sentence and started 300 ms before sentence-onset and continued for 300 ms after sentence-offset to give participants a chance to get used to the noise before the speech started. We used the unmasked recordings, with a 300 ms leading and trailing silence, as a control condition (“Quiet”).

### 2.3. Design

All experimental items were arranged in a Latin Square design to make 24 experimental lists of 90 sentences each. This length was chosen to be manageable in a single experimental session. On each list, all noise conditions as well as quiet were presented. This was done blocked by noise with 30 items per block, starting with either babble noise or white noise. This order was counterbalanced across participants. Half of them started with white noise, the other half with babble noise. Quiet was presented last to make the manipulations in the experiment less obvious to participants. In each block of noise, there was the same number (*N* = 15) of high- and low predictability items, presented in random order. Participants heard only one item of a pair, and only in one of the noise conditions. Each list started with a short practice block of four items, during which all types of noise as well as quiet were presented.

### 2.4. Procedure

**Figure 1:**
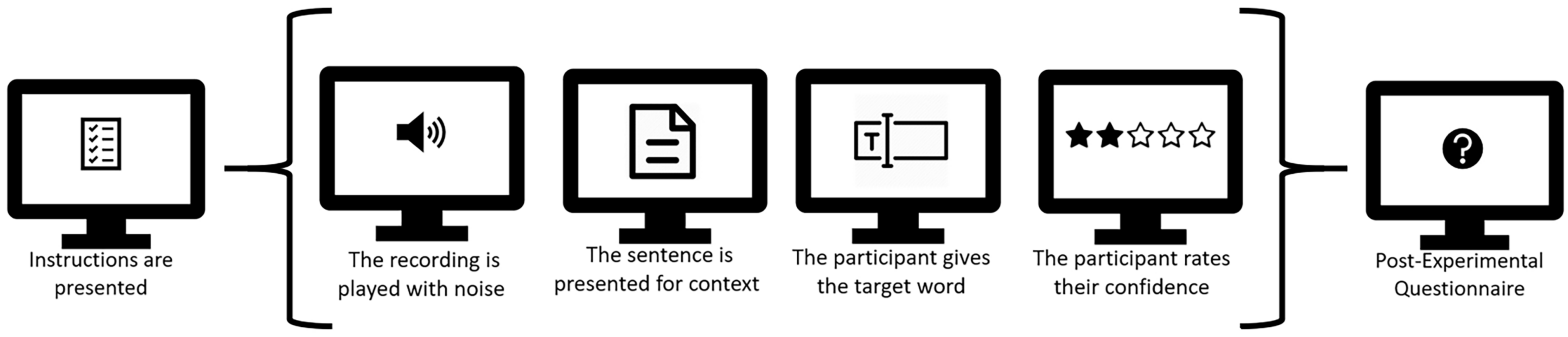
The different stages of the experiment, with a single trial between brackets. Participants completed 4 practice trials and 90 experimental trials (half of 180 to keep the experiment at a manageable length).

The experiment was run online, and hosted on Lingoturk, a crowdsourcing client (Pusse et al., 2016). Participants were instructed to complete the experiment on a computer (not a tablet or smartphone) in a quiet room. The experiment started with on-screen instructions of the task. These instructions included a sound check so that the participant could make sure the audio was working correctly before the experiment started. They were instructed to set the audio to a comfortable level. Due to the online setting of the experiment, we were unable to control the type of audio hardware participants used. In the post-experimental questionnaire we did include questions on how loud the participants’ testing surroundings were and if they were doing any secondary tasks (watching tv, texting, etc.), to get an idea of the conditions during the experiment. Participants first listened to a recording of a sentence while looking at a fixation cross. The length of the audio recordings ranged from 1,932 to 9,632 ms. After the item played, the screen automatically moved from the fixation cross to the next screen without delay. Participants then were asked to type in the final word they had heard on the next screen. Here, we presented the sentence (minus the sentence-final target word) in written form on the screen to ensure that the participant could use the contextual information even in difficult noisy conditions. Participants typed their response in a text box, and could start as soon as the screen with the sentence context and text box appeared. On the same screen, there was a question regarding their confidence in giving the correct response with a four-point scale. The next item’s recording started playing automatically as soon as the participant had clicked on ‘Next’ to go to the next trial. The task was not timed, so participants could take as long as they needed to make their responses. Figure 1 presents a schematic overview of the experiment.

### 2.5. Analyses

All participants’ responses were automatically classified on whether it was the *target* (the word that was played in the audio, e.g., in example 1A in Table 1 “Liege” / “lounger”), the similar sounding *distractor* (e.g., in 1A “Liebe” / “love”), or a different word entirely (e.g., in 1A “Platz” / “space,” *wrong*). The list of responses that were classified as wrong were then manually checked by the first author and a native German-speaking student assistant to correct misclassifications because of typos or spelling mistakes. In our statistical analyses, we added the trial number within each noise block (1–30 for 30 trials per block) as a variable to check for learning effects in the experiment.

To get a better idea of what information participants relied on when making their wrong responses, we coded the semantic fit of the incorrect responses (fitting or not fitting). Fitting responses resulted in a grammatical and meaningful sentence, as judged by a native German student-assistant. We also coded the phonetic distance between the incorrect responses and target items. We made phonetic transcriptions based on the Deutsches Aussprachewörterbuch (German Pronunciation Dictionary; Krech et al., 2009) and calculated the weighted feature edit distance using the Python package *Panphon* (Mortensen et al., 2016). This distance was normalized by dividing it by the longest of the two compared words. The normalized distance fell between 0 and 1.

## 3. Results

In our statistical analyses, we used generalized linear mixed models with logit link (GLMMs), implemented in the lme4 package (Bates et al., 2015) in R (R Development Core Team). These models allow both fixed and random effects, letting us control for variation on the participant- and item-level (Baayen et al., 2008; Barr et al., 2013). To improve convergence, all models were run using the bobyqa optimizer and increased iterations to 2·10^5^. Model comparisons were made to guide model selection based on the Akaike Information Criterion (AIC), models with the lowest AIC are reported below. We used forwards Helmert contrast coding for the Noise variable, so that the first contrast showed the difference between the Quiet condition and the mean of both types of noise (with weights of −1, 0.5, 0.5), and the second contrast showed the difference between Babble Noise and White Noise (using weights of 0, −0.5, 0.5). The other categorical predictor variables are treatment coded.

We first ran a model that included *Noise* and *Predictability*, as well as their interaction. *Noise* is a categorical predictor with three levels that was contrast coded using forwards Helmert coding, as explained above. *Trial Number* was included as a continuous predictor that was scaled. In order to use logistic regression, we collapsed *wrong* and *distractor* responses in our models and compare them to the *target* responses. Running all models with *targets* vs. *distractor* responses (leaving out *wrong* responses, which occur least), leads to very similar results. The model included random intercepts for Participant and Item, with random slopes of Noise and Predictability for both Participant and Item. The model revealed a significant effect of Predictability (*β* = −4.19, SE = 0.50, *z* = −8.43, *p* < 0.001), showing fewer target responses in the low predictability items. There was a significant effect for the first contrast of Noise (*β* = −1.91, SE = 0.61, *z* = −3.16, *p* < 0.01), showing a lower amount of target responses in noise compared to quiet. The interaction of Predictability and Noise was also significant (*β* = −2.02, SE = 0.41, *z* = −4.87, *p* < 0.001), showing a larger negative effect of noise on correct target responses in low predictability items than high predictability items. The other effects were not significant, as can be seen in Table 2.

**Table 2:** Model outcomes for the overall model (predictability * noise interaction).

	Estimate	SE	*Z*-Value	*p*-Value	
Intercept	5.07	0.52	9.52	<0.001	***
Predictability (LP)	−4.19	0.50	−8.43	<0.001	***
Noise Contrast 1	−1.91	0.61	−2.16	<0.01	**
Noise Contrast 2	−0.03	0.35	−0.09	0.93	
Trial Number	−0.10	0.07	−1.56	0.12	
Predictability (LP) : Noise Contrast 1	−2.02	0.41	−4.87	<0.001	***
Predictability (HP) : Noise Contrast 2	0.17	0.40	0.42	0.67	

*p < 0.05; **p < 0.01; ***p < 0.001.

**Figure 2:**
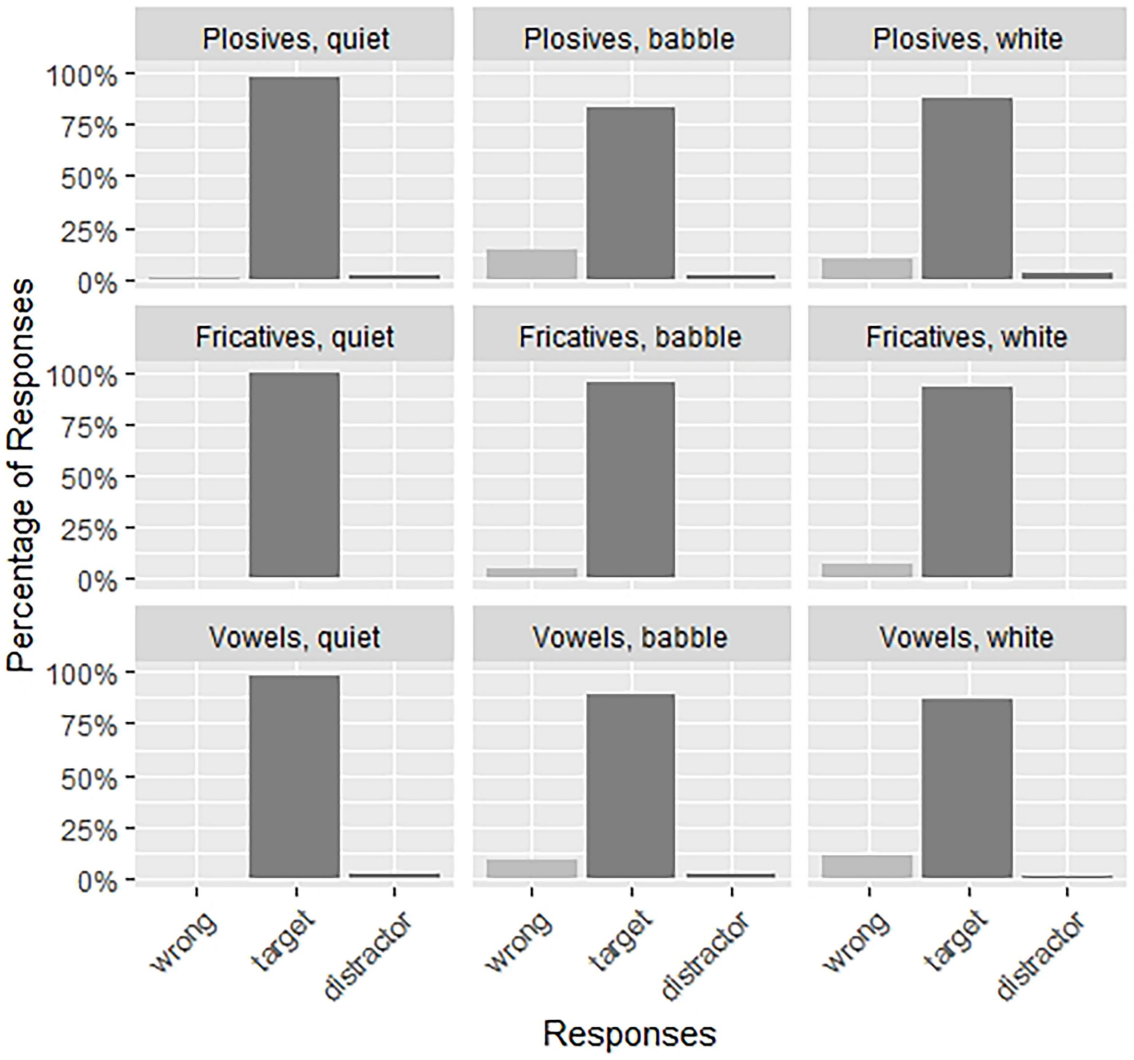
The proportion of participants’ responses (wrong, target, and distractor) for each of the three noise conditions (quiet, babble, white noise) and three sound types (plosives, fricatives, vowels) for the high predictability condition.

**Figure 3:**
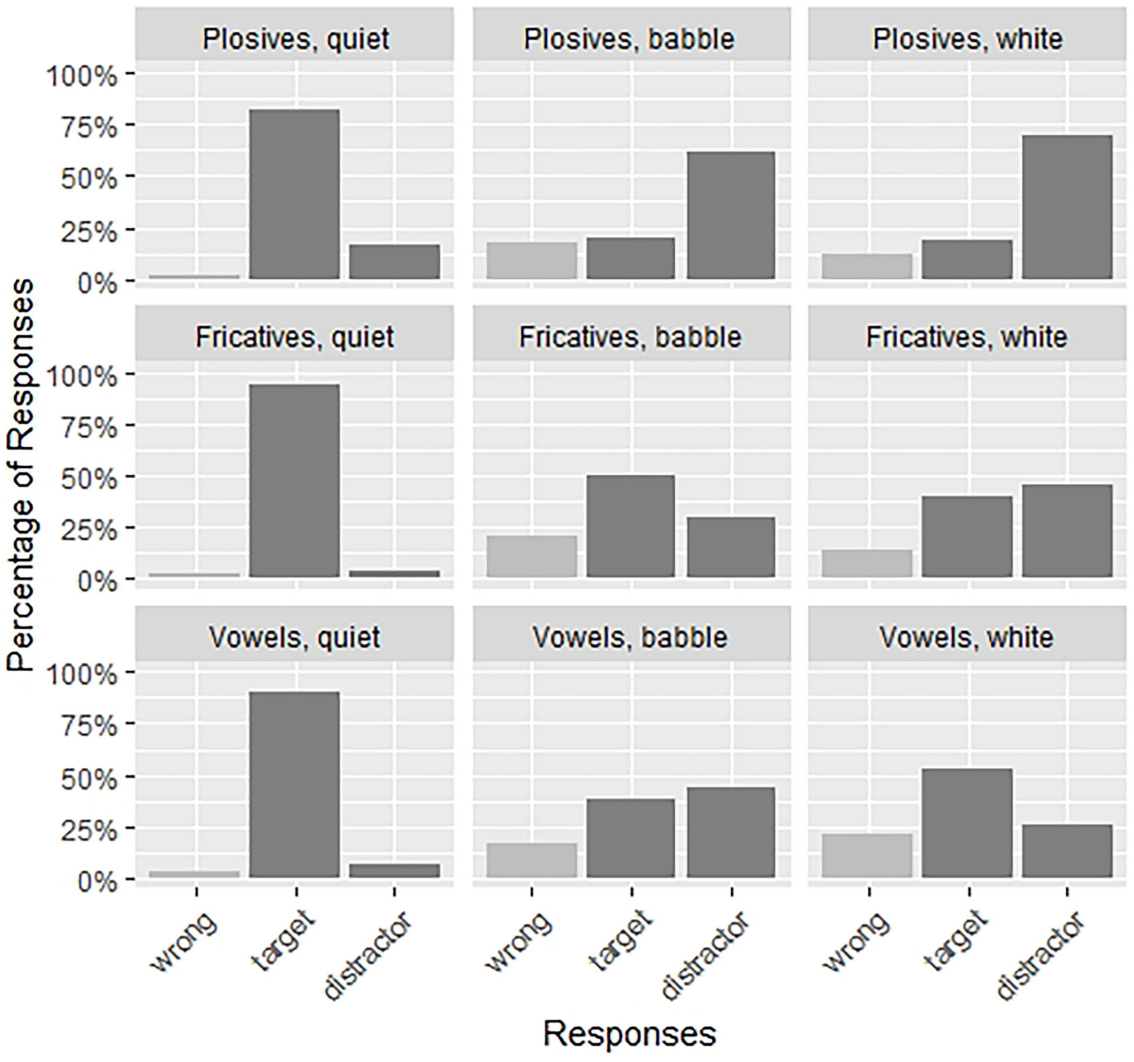
The proportion of participants’ responses (wrong, target, and distractor) for each of the three noise conditions (quiet, babble, white noise) and three sound types (plosives, fricatives, vowels) for the low predictability condition.

In the following analyses, we will analyze the subset of Low Predictability items in detail, as here we see interesting effects. In the High Predictability condition we find ceiling effects (see Figure 2). Reducing the size of the model also has the benefit of reducing the number of comparisons, which eases the interpretation of the model results. This means that per noise condition, a total of fifteen LP trials was analyzed for each participant. Figure 3 shows the responses for the low predictability items. We expect to find an interaction of Noise and Sound Contrast, which would indicate that the type of background noise has a different effect on intelligibility for the different speech sound contrasts in our stimuli.

We included three predictors in the LP model: *Noise*, *Sound Contrast*, and *Trial Number*. *Sound Contrast* is a categorical predictor with three levels, with Plosives as the base level. We additionally included the interaction of *Noise* and *Sound Contrast*. *Trial Number* is a continuous predictor that was scaled like before. The model includes random intercepts for Participant and Item, with a random slope of Noise for Participants. Including additional random slopes resulted in non-convergence.

The model revealed a significant main effect of *Noise*, but only for the first contrast indicating the difference between Quiet and both noise conditions together, showing that the noise conditions led to fewer target responses than Quiet (*β* = −3.29, SE = 0.24, *z* = −13.53, *p* < 0.001). There was no difference in the second contrast indicating the difference between Babble and White Noise (*p* = 0.80). However, recall that Plosives is the base level and that thus these main effects only hold for Plosives. We additionally found interactions of Noise and Sound Contrast when comparing Plosives to Vowels (*β* = 0.62, SE = 0.29, *z* = 2.13, *p* < 0.05 for Noise contrast 1 and *β* = 0.95, *SE* = 0.40, *z* = 2.39, *p* < 0.05 for Noise contrast 2), but not when comparing Plosives to Fricatives (*p* > 0.12). These results suggest different effects of Noise depending on the Sound Contrast, see Table 3. The model also showed a main effect of Sound Contrast, with more target responses for both Fricatives and Vowels compared to Plosives (*β* = 1.88, SE = 0.29, *z* = 6.60, *p* < 0.001 for Fricatives, and *β* = 1.81, SE = 0.28, *z* = 6.36, *p* < 0.001 for Vowels). As can be seen in Table 3, the other effects were not significant.

**Table 3:** Model outcomes for the overall model (low predictability subset).

	Estimate	SE	*Z*-Value	*p*-Value	
Intercept (sound contrast plosives)	−0.71	0.25	−2.8	0.01	**
Noise Contrast 1	−3.29	0.24	−13.53	<0.001	***
Noise Contrast 2	−0.09	0.34	−0.26	0.80	
Sound Contrast Vowels	1.88	0.29	6.6	<0.001	***
Sound Contrast Fricatives	1.81	0.28	6.36	<0.001	***
Trial Number	0	0.07	0.07	0.95	
Noise 1 : Sound Contrast Fricatives	0.2	0.3	0.65	0.52	
Noise 2 : Sound Contrast Fricatives	−0.6	0.39	−1.53	0.12	
Noise 1 : Sound Contrast Vowels	0.62	0.29	2.13	<0.05	*
Noise 2 : Sound Contrast Vowels	0.95	0.4	2.39	<0.05	*
Sound Contrast Vowels vs Fricatives	−0.07	0.28	−0.26	0.80	

*p < 0.05; **p < 0.01; ***p < 0.001.

In order to compare Fricatives to Vowels, we rerun the same model with Fricatives as the base level for *Sound Contrast*. This model showed no significant difference between Fricatives and Vowels (*p* = 0.80).

To get better insight into the interaction of Sound Contrast and Noise, we next turn to the low predictability subsets of Plosives, Vowels, and Fricatives. These analyses will show exactly how the two types of background noise affected each of the contrasts. We expect in particular an adverse affect of white noise for fricatives, and an adverse effect of babble noise for vowels. For each subset, we ran a GLMM that only differed in its random structure, as indicated below. Again, Noise is contrast coded using forward Helmert coding. The first contrast shows the difference between Quiet on one hand and both noise types on the other, and the second contrast shows the difference between Babble Noise and White Noise. Results for the three models are presented in Tables 4–6 for the subsets of plosives, fricatives, and vowels, respectively.

**Table 4:** Model outcomes for the subset of plosives.

	Estimate	SE	*Z*-Value	*p*-Value	
Intercept	−0.7	0.25	−2.82	<0.01	**
Noise Contrast 1	−3.15	0.28	−11.29	<0.001	***
Noise Contrast 2	−0.05	0.29	−0.16	0.87	
Trial Number	−0.11	0.13	−0.86	0.39	

*p < 0.05; **p < 0.01; ***p < 0.001.

**Table 5:** Model outcomes for the subset of fricatives.

	Estimate	SE	*Z*-Value	*p*-Value	
Intercept	1.4	0.39	3.55	<0.001	***
Noise Contrast 1	−3.67	0.56	−6.58	<0.001	***
Noise Contrast 2	−0.85	0.35	−2.42	<0.05	*
Trial Number	0.13	0.15	0.88	0.38	

*p < 0.05; **p < 0.01; ***p < 0.001.

**Table 6:** Model outcomes for the subset of vowels.

	Estimate	SE	*Z*-Value	*p*-Value	
Intercept	2.04	0.66	3.08	<0.01	**
Noise Contrast 1	−4.58	1.26	−3.62	<0.001	***
Noise Contrast 2	0.76	0.25	3.08	<0.01	**
Trial Number	−0.01	0.12	−0.1	0.92	

*p < 0.05; **p < 0.01; ***p < 0.001.

The model for the LP subset of Plosives (all LP trials in which the minimal pair had a plosive contrast: *N* = 744) included two predictors, Noise and Trial Number (scaled as before). There are random intercepts for Participant and Item, but inclusion of random slopes led to non-convergence and singular fit. We find only a significant effect of the first Noise contrast, so between Quiet and both types of Noise, with fewer target responses in Noise than Quiet (*β* = −3.15, SE = 0.28, *z* = −11.29, *p* < 0.001). The lack of a significant difference for the second contrast (*p* = 0.87) suggests that there is no difference between Babble Noise and White Noise in the effect they have on the recognition of the target words. As can be seen in Figure 3 in two right-most panels on the top row, there is indeed no large difference in the amount of target responses depending on the type of noise.

For the LP subset of Fricatives (all LP trials in which the minimal pair had a fricative contrast: *N* = 719) we included the same two predictors, Noise and Trial Number, with the same random intercepts of Participant and Item as for the model for Plosives. This model contains an additional random slope of Noise for Participant. The model reveals both a significant adverse effect of Noise compared to Quiet (Contrast 1: *β* = −3.67, SE = 0.56, *z* = −6.58, *p* < 0.001), and a significant adverse effect of White Noise compared to Babble Noise (Contrast 2: *β* = −0.85, SE = 0.35, *z* = −2.42, *p* < 0.05). These results suggest that unlike Plosive items, Fricative target pairs are more strongly affected by White Noise than Babble noise. This can clearly be seen in Figure 3: While in Babble Noise (depicted in the middle panel of the figure) most responses are target responses, in White Noise (right-most panel on the middle row) there are more distractor responses than target responses, showing the difficulty of recognizing fricatives in this type of noise.

Finally, the model for the LP subset of Vowels (all LP trials in which the minimal pair had a vowel contrast: *N* = 696) also included the same two predictors as before, Noise and Trial Number. It had random intercepts for Participant and Item, with a random slope of Noise for Item. There was a significant effect of Quiet vs. Noise (*β* = −4.58, SE = 1.26, *z* = −3.62, *p* < 0.001) with more target responses in Quiet. Additionally, the second contrast also showed a significant effect (*β* = 0.76, SE = 0.25, *z* = 3.08, *p* < 0.01), showing more target responses in White noise compared to Babble noise, an effect in the opposite direction as for Fricatives. Again, this is visible in Figure 3, where we see a majority of distractor responses in Babble Noise (middle panel on the bottom row), while in White Noise (right-most panel on the bottom row) there is a majority of target responses, showing this is the easier condition to recognize vowels correctly.

To analyze which kind of information participants relied on when making their incorrect responses – top-down predictions or bottom-up auditory processes – we coded the semantic fit and phonetic distance to the target word for all wrong responses (*N* = 396; both from the HP and LP condition). Empty responses (where participants typed only *?* or *-*, for example, *N* = 5) were removed in this analyses. If participants rely more on semantic context to give their answer, we expect that their response fits the sentence context and perhaps has a higher phonetic distance to the target. If, on the other hand, they rely more on the sound signal, we expect that the phonetic distance to the target is smaller, but that the word might not fit the semantic context. We have only analyzed this for the wrong responses, as for the targets and distractors, these values are a given: In HP items, the distractor never fits the sentence, while in LP the target never fits the sentence semantically, and they have a similar phonetic distance.

**Figure 4:**
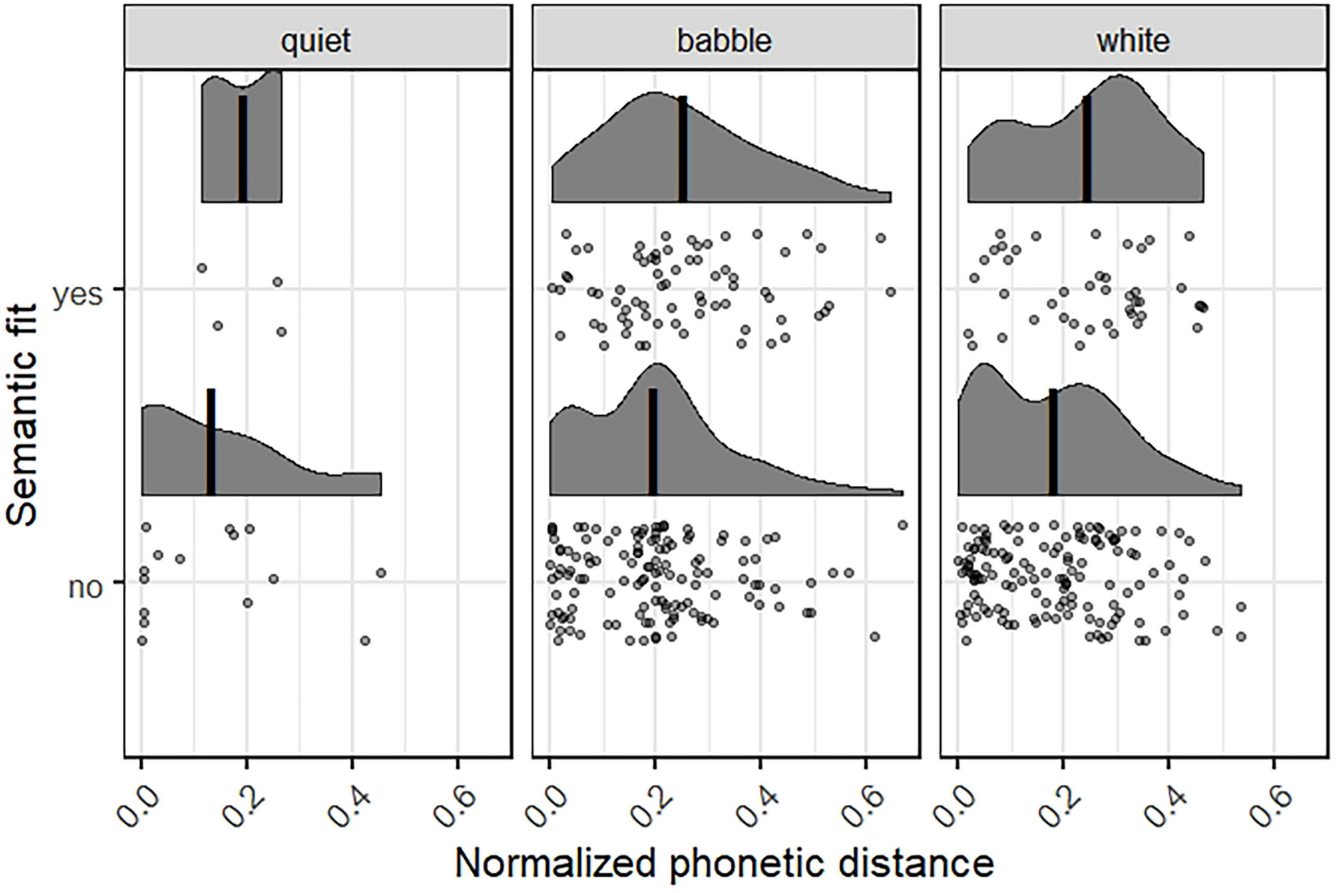
The wrong responses that semantically fit or did not fit the sentence, plotted with the normalized phonetic distance, in each of the three noise conditions. Lower phonetic distance means more similar to the target item. The vertical black lines show the mean phonetic distance for each condition. Each dot represents a single wrong response, the shaded curves show the density plots for these.

These results are presented in Figure 4, showing the semantic fit, yes or no, and normalized phonetic distance to the target word for each of the three noise conditions. Lower normalized phonetic distance scores mean that the participant’s wrong response sounds more like the target. We see that for all noise conditions, most responses did not fit the sentence semantically: 15 vs. 4 for Quiet, 128 vs. 71 for Babble Noise, and 135 vs. 43 for White Noise. The density peaks of the phonetic distance distributions as well as the mean values (shown by the vertical lines in Figure 4) are lying more toward the lower distances for those responses that do not fit the sentence context. This suggests a trade-off between acoustic fit and semantic fit: Participants made their response based on what they heard at a cost of fitting the semantic context.

In the experiment, we additionally asked participants to rate their confidence in giving the correct response. We will not cover the results of the confidence ratings here, but these can be found in the Supplementary Material.

## 4. Discussion

The present study investigated how during speech comprehension multiple sources of information are combined by the listener, filling a gap in the empirical literature that has not combined context, types of background noise, and a systematic manipulation of different types of speech sounds in a single experiment. We examined mishearings occurring when listening to speech in background noise, as a function of the predictability of the context and certain sound characteristics of the target word. We were particularly interested in the interaction of background noise type (white noise or multi-speaker babble noise) and the sound contrast in the stimuli (minimal pairs of plosives differing in the place of articulation, tense/lax vowel pairs, and pairs of voiceless (af)fricatives). We expected that, based on previous literature, the plosives would be difficult to recognize correctly in background noise in general, while for fricatives and vowels this depends on the type of background noise (fricatives have energy in the same frequency bands as white noise, Phatak et al., 2008; while the formant values of vowels are easily lost in fluctuating babble noise, Pickett, 1957; Gordon-Salant, 1985; Benkí, 2003; Weber and Smits, 2003). This was confirmed in our study. In the high predictability condition, both the audio and the presented written sentence context point to the target, while in the low predictability condition the context supported the distractor response. Thus, finding distractor responses in the low predictability subset shows us that participants relied on the semantic context. Of course, as the target and distractor form minimal pairs, the speech signals for both words overlap greatly.

As expected, we find a main effect of noise, with more correct responses in quiet than in either type of background noise. This can be seen as a control condition, replicating various previous experimental findings (Kalikow et al., 1977; Gordon-Salant, 1985; Phatak et al., 2008; Van Os et al., 2021). Differences between babble and white noise occurred in interaction with the type of sound contrast (for detailed discussion, see below). Previous experiments showed conflicting results on whether babble noise or white noise disrupts recognition of speech most (e.g., Horii et al., 1971; Gordon-Salant, 1985; Taitelbaum-Swead and Fostick, 2016). The results from the present study suggest that the effect of the two types of noise is strongly dependent on the characteristics of the stimuli. This might partially explain the conflicting results in previous literature on this topic, besides other factors like the task, noise levels, and the tested population.

We do not find any significant effects of trial number in our data, suggesting there was no learning effect. Previous research (Van Os et al., 2021) did report a learning effect with more correct answers as the blocks of the experiment went on, indicating that participants learned to rely less on the contextual information. Listeners have been found to re-weight acoustic and contextual cues based on their statistical properties (Bushong and Jaeger, 2019a). It might be the case that in the present experiment, participants took longer to get used to the different background noise types and did not have time within each block to re-weight the information from different sources (Van Os et al., 2021 used only a single type of background noise).

We additionally expected to find an interaction of background noise and the items’ predictability. This expectation was confirmed by the data. The results provide evidence for the role of the bottom-up acoustic signal, as shown by the ceiling effect in the quiet listening condition also in low predictability items, as well as evidence for the role of the top-down signal, shown by the ceiling effects in the noisy high predictability items. The interaction between predictability and background noise in our data (as well as the interaction between speech sound and background noise type; see below) shows that these two types of evidence are rationally combined, as predicted by models of speech comprehension (e.g., Oden and Massaro, 1978; Luce and Pisoni, 1998; Norris and McQueen, 2008). Participants rely on the information that is most available to them in a particular experimental condition. Previous studies found that sentence context affects the interpretation of phonemes, biasing these to fit the semantic context (Spencer and Halwes, 1981; Miller et al., 1984; Connine, 1987). Later work found that this effect of context is present even when the biasing context occurs after the ambiguous sound (Connine et al., 1991). Bushong and Jaeger (2017) replicated this finding that listeners have the ability to maintain acoustical information throughout the sentence, but prefer to respond before the relevant context has been presented. Comparing behavioral data to several models, Bushong and Jaeger (2019b) found that ideal integration models provide a good fit of human listeners’ behavior, who maintain detailed acoustic representations.

We also see this trade-off between semantic context and acoustic signal when looking at the subset of wrong responses (those responses that were neither the target nor the distractor word from the minimal pair; *N* = 369). We coded the semantic fit to the target sentence context as well as the phonetic distance to the target word, and found that when the wrong response does not fit the semantic context, the phonetic distance to the target word is smaller, meaning the words sound more alike. In our experiment, participants, when making a wrong response, based this on the speech signal at a cost of fitting the semantic context. This is interesting, as we presented participants with the written context on the screen. It would have been very easy for them to rely on this information, despite being asked to focus on the speech. It might be the case that over the course of the experiment, participants learned to rely less on the written context, having realized it can be misleading. Of course, this analysis is based on a small subset of the data. Overall, participants tended to rely on the acoustic signal in quiet listening conditions and more on the semantic context when listening conditions were more difficult.

The level of spectral overlap between noise and phoneme should affect how much listeners rely on the sentence context: this is stronger in higher levels of noise. In the present study, we manipulated the amount of overlap between the speech and noise signals by using combinations of different types of background noise and speech sound contrasts. Results followed our predictions. Plosive pairs are the most difficult speech sound (out of the ones tested in this study) to be recognized correctly, and they do not show a difference between the two types of background noise. We find a difference between babble noise and white noise for the fricatives, whose recognition is impaired in white noise compared to babble noise, and for the vowels, which show the opposite pattern compared to fricatives. A previous study found many errors for fricatives in babble noise (Weber and Smits, 2003). These errors were found to be of varying kinds, namely manner, place, and voicing errors. In the stimuli of the present study, the most likely error was a place error, as the minimal pairs were constructed to differ only in place of articulation, keeping other features the same. Therefore, we would expect fewer errors in the recognition of fricatives in babble noise, as the chance of some errors is reduced. While participants could respond from an open set of candidate words (as it was not a multiple choice task), they most often responded with the distractor rather than a different word.

The Noisy Channel Model (Shannon, 1949; Levy, 2008; Levy et al., 2009) is a model of human sentence comprehension under noisy input. Typical language is seldom free of noise: among other sources, there can be environmental noise, a low effort at clear communication on the part of the speaker, or insufficient attention on the part of the comprehender. The Noisy Channel Model proposes that language comprehension is equipped to deal with this noise by using Bayesian decoding of the intended meaning. Language comprehension is a rational process, where all different sources of information that are available to the listener are combined. Human language processing is designed to handle the task of recovering a speaker’s intended meaning even in noisy utterances. It relies in this on prior knowledge in the form of linguistic and world knowledge (which meanings are more plausible; what is the base-rate frequency of certain grammatical constructions), as well as knowledge about what the most likely corruptions due to the noise might be. Using these two types of information and Bayes’ Rule, the probability that a certain sentence was intended by the speaker given the perceived sentence can be calculated.

Gibson et al. (2013) tested predictions made by the Noisy Channel Model in a study investigating the interpretation of syntactic alternations. They used different English constructions (active / passive, subject /object locative, transitive/intransitive, double object/prepositional phrase object) in a plausible and implausible version. The difference between these two versions was expressed in the number of insertions and deletions of words. Gibson et al. then presented these items in written form to participants with comprehension questions that were used to determine the participants’ interpretation of the experimental item. Participants preferred a plausible interpretation over the literal one, but this was dependent on the number of edits (insertions and deletions). The larger the number of edits, the more likely it was that the sentence was interpreted literally. When the perceived noise rate increased through the inclusion of filler items with syntactic errors, participants more often interpreted the sentence in its plausible meaning rather than literally. Taken together, the results of this study provide strong evidence in favor of the Noisy Channel Model in sentence comprehension. These results have been replicated using the same method (Poppels and Levy, 2016), who also found that comprehenders consider positional exchange of function words when interpreting implausible sentences, and using a different method where participants were asked to retype the experimental item and edit if they thought there were mistakes (Ryskin et al., 2018). However, these studies made use of written materials that participants could read multiple times if they wanted, and as such is different from speech comprehension, during which the short-lived speech signal is presented only once and cannot be listened to again. Using the same materials but in auditory form, Gibson et al. (2016) and Gibson et al. (2017) found similar results providing evidence for the Noisy Channel Model.

In their original study, Gibson et al. (2013) quantified the edit distance between the plausible and implausible version of their alternations in a change consisting of insertions and deletions of function words (see also Gibson et al., 2016, 2017). The present study changed the type of background noise to manipulate the distance between the target and distractor. Here the distance between the two depends on the similarity between the acoustic signal of the speech sound and that of the background noise. As such, it is better grounded in the strength of masking of the signal, and less arbitrary than the edit distance measured in terms of insertions and deletions. Using auditory stimuli of a different type than syntactic alterations, we have found support for the Noisy Channel Model’s predictions in a more naturalistic setting.

A theory that stands in contrast to algorithmic computations, such as rational Bayesian models like the Noisy Channel Model, is the Shallow Processing account, also known as “good-enough processing” (Ferreira et al., 2002; Ferreira, 2003; Ferreira and Patson, 2007). It states that language comprehension relies on heuristic processing as well as algorithms, generating superficial interpretations of sentences that can in fact be inaccurate. Two heuristics proposed by Ferreira (2003) are based on plausibility and word order. The first studies investigating this theory based their evidence on thematic role assignment in active and passive sentences, as well as subject- and object cleft sentences and garden-path sentences, and argued that the use of “good-enough representations” is common in language processing in general (Ferreira et al., 2002; Ferreira, 2003). The representations based on shallow processing are often good enough for everyday communication (as opposed to being tested in psycholinguistic experiments), the most common task that listeners perform, leading only occasionally to misunderstandings (Ferreira, 2003; Christianson, 2016). Through shallow processing, comprehension can be fast enough to keep up with dialog and takes as little effort as possible. Using plausibility-based heuristics in shallow processing is generally quicker than using syntactic algorithms, and it might also be a strategy to conserve available resources, especially for older adults (Ayasse et al., 2021).

The Noisy Channel Model and Shallow Processing are similar in multiple aspects and share some central ideas such as processing guided by context and at times incorrect final interpretations (Traxler, 2014; Christianson, 2016; Kuperberg and Jaeger, 2016), and both explain how misinterpretations during speech comprehension can occur. Previous papers have made suggestions to link (Bayesian) predictive models and the Shallow Processing account (Ferreira and Lowder, 2016). They do this through the notion of information structure, distinguishing given and new information. Following Haviland and Clark (1974), they state that given information has already been introduced and stored away in long-term memory, while new information needs to be integrated with this knowledge. This information can come from various sources: lexical, syntactic, semantic, or pragmatic. Ferreira and Lowder (2016) suggest that only the part of the sentence that presents given information is processed shallowly, while the rest of the sentence is processed following (Bayesian) algorithms. As there is a tendency to present given information before new information, comprehenders would assume this is the case, and process the beginning of the sentence shallowly, while processing the latter part deeply (Ferreira and Lowder, 2016). In psycholinguistic experiments, like the current one, usually each item is a separate sentence without context provided by a longer discourse. Thus, there is a hardly any information structure on the discourse level when interpreting these separate sentences, which according to this theory would mean that there would be no shallow processing in these experiments. Especially in the present study, the critical part is sentence-final and should be processed following Bayesian algorithms rather than through shallow processing. It is unclear what predictions are made by Shallow Processing in psycholinguistic experiments where each item consists of an unrelated sentence. Additionally, as mentioned above, Shallow Processing might not line up to the goals of psycholinguistic experiments as these can differ from those in everyday language use. It should be clarified exactly when and where Shallow Processing takes place, so that it can be contrasted to other approaches and directly tested in experiments.

Kuperberg and Jaeger (2016) argued that shallow processing may arise from prediction, such that highly constraining sentence contexts which give rise to strong anticipations should lead to shallow processing. This would fit with our high predictability condition: The prediction in this case would be that misinterpretation of the sentence-final word would be common in the low predictability condition in the present experiment, also in quiet. Here it contrasts with predictions made by the Noisy Channel Model, and, crucially, also the results of the present study. In the low predictability condition in quiet, we find target responses close to ceiling, particularly for fricatives and vowels, showing that participants were relying on the acoustic signal rather than the (misleading) sentence context.

Furthermore, the Shallow Processing account does not make detailed predictions about the different noise and sound contrast conditions in the present experiment. In particular, Shallow Processing does not depend on the clarity of the signal, like the Noisy Channel Model does. Therefore, there are no specific predictions about any possible interaction effects of noise type and speech sound contrast. In order to compare the Noisy Channel Model and the Shallow Processing account in experiments such as the present one, the Shallow Processing account should be extended so that it makes predictions about this interaction and specifies how the clarity of the (acoustic) signal influences processing depth.

Another line of research that investigates how bottom-up and top-down processes interact is that of local coherence effects (e.g., Konieczny et al., 2009; Kukona and Tabor, 2011; Kukona et al., 2014). A sentence like “The coach smiled at the player tossed a Frisbee by the opposing team” contains a locally coherent phrase “The player tossed a Frisbee.” Using various methods, studies have shown that these locally coherent phrases influence sentence processing, leading to longer reading times and error detection times (Tabor et al., 2004; Konieczny, 2005; Konieczny et al., 2009). This suggests that participants actively considered the phrase “The player tossed a Frisbee” even though it is incompatible with the earlier parts of the sentence. The work on local coherence shows that the bottom-up cues in language comprehension get integrated as well, even if they are inconsistent with the top-down predictions of the sentence structure. Thus, top-down predictions do not rule out bottom-up perceptions, people engage in an interpretation that is locally coherent, even though this interpretation is incompatible with the prior context. In the current experiment, this would mean that in the LP condition, participants consider the target response even if they respond with the distractor word in the end (if the word was sufficiently audible). Future research could investigate how the information from the acoustic signal and the semantic context get integrated *online* in an experiment similar to the one reported here.

### 4.1. Limitations

One limitation of the current study is its online design. While it allowed us to collect data in lock-downs during the Covid-19 pandemic, it also means we were unable to control the audio setting as we would have been able to in a lab study. We could not collect hearing thresholds of participants and had to rely on self-reported hearing issues. It is possible that some participants were unaware of existing problems with their hearing. Furthermore, we could not control the equipment participants used to play the audio in the experiment, nor the level they set or the amount of background noise in their surroundings. By using instructions and a post-experimental questionnaire, we tried to get an idea of these factors for each participant. Still, this source of variation due to the online design might have affected our results.

While we tested three different categories of speech sounds (plosives, fricatives, vowels), we did not control for possible variation in recognition within each category. Previous studies have found that coarticulation effects play a role in recognition of speech sounds, in particular consonants (Gordon-Salant, 1985; Alwan et al., 2011). In our study, we were not able to control the direct phonetic context of the minimal pair contrasts to minimize these effects. Studies have also found that within each type of sound contrast, some sounds might be more robust to noise than others. For example, ʃ (as in ship) has been found to be easier to recognize than other voiceless fricatives (Gordon-Salant, 1985; Weber and Smits, 2003). For vowels, the second formant values are most strongly obscured in background noise (Parikh and Loizou, 2005). We used pairs of tense and lax vowels, but for these sounds there are larger differences in the second formant for the back vowels than for most front vowels (Hoole and Mooshammer, 2002). This leads to differences in recognition or adverse effect of noise within our sound contrast categories. Here, the unbalanced contrasts within each category (plosives, fricatives, vowels) might have affected the results, as it is possible that the interference of noise type is stronger for some of the contrast pairs, that might have occurred more or less in our stimuli as a whole. An additional factor here is that our experiment is limited to having a single female speaker. With speakers of the same sex with different speech/voice characteristics or speakers of a different sex, the results might differ. The voices of these speakers might interact slightly differently with the noise types, affecting which sounds are recognized better or worse. The experiment should be replicated with different speakers to test for more robust effects.

Other factors have been found to affect word recognition, such as the lexical status of the word (real word vs. non-word), word frequency, length, and neighborhood effects. Due to the way we constructed our stimuli, we were not able to carefully control our items for these factors.

## 5. Conclusion

During speech comprehension, multiple sources of information are available. Major models of speech comprehension (e.g., FMLP, Oden and Massaro, 1978; NAM, Luce and Pisoni, 1998; Shortlist B, Norris and McQueen, 2008) already combine multiple sources of information. However, these models are often based on empirical data that is based on mono-syllabic word recognition rather than full sentences or larger contexts. Previous studies that investigated predictability effects in noise did not carefully control the types of sounds and how they are affected by noise (Kalikow et al., 1977; Boothroyd and Nittrouer, 1988; Hutchinson, 1989; Pichora-Fuller et al., 1995; Sommers and Danielson, 1999; Dubno et al., 2000), while the literature on effects of background noise on speech sounds does not specifically manipulate predictability effects in sentence comprehension (Pickett, 1957; Gordon-Salant, 1985; Phatak et al., 2008; Cooke, 2009; Alwan et al., 2011). Additionally, results on the effect of background noise are inconclusive regarding which type of noise affects comprehension most severely (Horii et al., 1971; Danhauer and Leppler, 1979; Gordon-Salant, 1985; Nittrouer et al., 2003; Taitelbaum-Swead and Fostick, 2016). We addressed this gap by presenting an experiment that investigates how different sources of information are combined while manipulating the predictability of the context and the clarity of the acoustic signal. Our stimuli contain small differences in intelligibility by combining different types of background noise with different speech sound contrasts that are more or less strongly affected by that noise. Our results show that it is important to consider the effect of the type of noise masker. Listeners use all the cues that are available to them during speech recognition, and these cues crucially depend on the masking noise in the background. In this process, listeners are rational and probabilistically combine top-down predictions based on context with bottom-up information from the acoustic signal, leading to a trade-off between the different types of information. This is in line with the idea of a rational listener based on Bayesian principles as suggested by multiple models of speech comprehension. Insights from this study might improve practical applications such as for speech synthesis systems.

## Data availability statement

The raw data supporting the conclusions of this article will be made available by the authors, without undue reservation.

## Ethics statement

The studies involving human participants were reviewed and approved by Deutsche Gesellschaft für Sprachwissenschaft (DgfS) ethics committee. The patients/participants provided their written informed consent to participate in this study.

## Author contributions

MO, VD, and JK were involved in the planning and designing of the study. MO analyzed the data and wrote all parts of the manuscript. VD and JK made suggestions to the framing, structuring, and presentation of the findings, as well as on the interpretation of the findings. All authors contributed to the article and approved the submitted version.

## Funding

This study was funded by the Deutsche Forschungsgemeinschaft (DFG, German Research Foundation) – Project-ID 232722074 – SFB 1102.

## Conflict of interest

The authors declare that the research was conducted in the absence of any commercial or financial relationships that could be construed as a potential conflict of interest.

## Publisher’s note

All claims expressed in this article are solely those of the authors and do not necessarily represent those of their affiliated organizations, or those of the publisher, the editors and the reviewers. Any product that may be evaluated in this article, or claim that may be made by its manufacturer, is not guaranteed or endorsed by the publisher.

## Footnotes

^1^This pretest experiment was run hosted on Lingoturk (Pusse et al., 2016) and run on Prolific (prolific.co). A set of 10 participants saw the experimental item on the screen and were asked to complete the sentence with a single word.

^2^ https://osf.io/9qbv4

^3^ http://bbcsfx.acropolis.org.uk/
