# Supplementary material for: Rational speech comprehension: Interaction between predictability, acoustic signal, and noise
Source: Front Psychol. 2022 Dec 16;13:914239. doi: 10.3389/fpsyg.2022.914239 (PMC9802670; doi:10.3389/fpsyg.2022.914239)
Supplement: Supplementary file 1 [file Data_Sheet_1.PDF]

Supplementary Material

1. Analysis of Confidence Ratings

In the experiment, we asked participants on every trial to rate how confident they were in giving the correct response. For this, they had a four point scale, ranging from 1 (completely uncertain, guessed) to 4 (completely certain). Previous studies (Failes et al., 2020; Rogers et al., 2012; Sommers et al., 2015) reported *false hearing effects*, that is, increased confidence in giving the correct response even for incorrect responses when listening in noise. This effect has been found to be larger for older adults than younger adults, although a recent study (Van Os et al., 2021) found no evidence of false hearing for either group of participants. As the present study only tested younger adults, we do not expect to find effects of false hearing. Instead, we expect that confidence ratings will depend on the difficulty of the listening condition, and thus be lower in adverse conditions like background noise, or low predictability items.

Figure 1 presents the participants' responses for each of the predictability and response type conditions, split for the three noise levels. It is collapsed across the three speech sound types. It shows that the majority of responses in the high predictability condition are correct target responses, made with high confidence. In background noise, the number of wrong responses increases slightly, but these are rated with low confidence. In the low predictability condition, we find still many target responses in quiet, still with predominantly high confidence. In background noise, there is an increase in distractor responses, which are rated with high confidence. Again, wrong responses increase in noise, and are generally rated with low confidence.

In our statistical models, which are presented below (see Table 1), we have transformed these responses into a binary variable of low confidence (ratings 1 and 2) and high confidence (ratings 3 and 4). We used general linear mixed models (GLMM), implemented in the lme4 package (Bates et al., 2015) in R (R Development Core Team, 2022) to analyze our data. To improve convergence, all models were run using the bobyqa optimizer and increased iterations to $2*10^5$. We used a step-down approach to select the models, and used the Akaike Information Criterion (AIC) for model selection. The model with the lowest AIC are reported in Table 1. We used forward Helmert contrast coding for the Noise variable and for the Response Type variable. For Noise, this meant that the first contrast showed the difference between the Quiet condition and the mean of both types of noise, and the second contrast showed the difference between Babble Noise and White Noise. For Response Type, the first contrast compared correct Target responses to the mean of both types of incorrect (Distractors and Wrong) responses, while the second contrast compared Distractor responses and Wrong responses. Other categorical predictors were treatment coded.

The final model included fixed effects of Noise, Response Type, Predictability (categorical predictor with two levels, mapping the High Predictability condition on the intercept), and Trial Number (continuous predictor of the trial number in each block, scaled to improve convergence). The

model also included all two-way interactions between Noise, Response Type, and Predictability. Additionally, the model included random intercepts by Participant and by Item. Inclusion of random slopes led to singular fit of the model.

We find a main effect of Response Type (β = -2.34, *SE* = 0.19, *z* = -12.06, *p* < .001), showing that participants are less confident of incorrect (Distractor and Wrong) responses than correct Target responses. The difference between Distractor responses and Wrong responses did not reach significance as a main effect (*p* = .10). A significant main effect of Predictability (β = -0.89, *SE* = 0.21, *z* = -4.19, *p* < .001) shows that participants had lower confidence ratings in Low Predictable items. A significant main effect for the first Noise contrast (β = -1.26, *SE* = 0.34, *z* = -3.73, *p* < .001) shows that participants were less confident of their responses in background noise compared to quiet. The second Noise contrast (comparing Babble to White Noise) did not reach sigificance (*p* = .06). We find a significant interaction of Response Type and Predictability (β = 1.40, *SE* = 0.20, *z* = 6.93, *p* < .001), showing higher confidence ratings for incorrect responses in the Low Predictability condition. This is most likely due to the distractor responses, which did fit the semantic context in this condition, and would therefore be made with higher confidence. In the Low Predictability condition, the confidence ratings for wrong responses were lower than for distractor responses (β = -1.35, *SE* = 0.53, *z* = -2.56, *p* < .05), in line with this explanation. The significant interaction of Predictability and Noise suggests that confidence ratings were higher in noise than quiet in the Low Predictability condition (β = 0.79, *SE* = 0.34, *z* = 2.32, *p* < .05), again, most likely due to increased confidence in distractor responses in this predictability condition. Finally, there was a significant interaction effect of Response Type and Noise (β = 0.79, *SE* = 0.16, *z* = 4.88, *p* < .001), showing higher confidence ratings for incorrect (Distractor and Wrong) responses in Background Noise (Babble and White) compared to Quiet. Results for all effects can be found in Table 1.

Taken together, these results suggest that participants' confidence ratings depend on the difficulty of the listening condition and how much evidence they have to support their response. We see higher confidence ratings in quiet listening conditions, in particular when the response was a correct target one. In the high predictability condition, the confidence ratings are high for the two noise conditions as well. Here, despite the noise, both the provided sentence context and the acoustic signal point to the target, thus giving participants multiple sources to base their response on. This combination of information sources leads to higher confidence. When two sources of information are conflicting, as is the case in the low predictability condition, the number of responses as well as the confidence ratings drop in background noise for target responses, while they rise for distractor responses, which fit the semantic context. Wrong responses, which might fit either the semantic context or the acoustic signal (or neither) are rated with lower confidence. These results are in line with previous findings (Van Os et al., 2021).

Table 1

Model Outcomes for the Overall Model

	Estimate	SE	Z-value	p-value	
Intercept (Predictability High)	1.52	0.23	6.55	< .001	***
Response Type Contrast 1	-2.34	0.19	-12.06	< .001	***
Response Type Contrast 2	-0.87	0.52	-1.66	0.1	.
Predictability (Low)	-0.89	0.21	-4.19	< .001	***
Noise Contrast 1	-1.26	0.34	-3.73	< .001	***
Noise Contrast 2	-0.39	0.21	-1.96	0.06	.
Trial Number	-0.08	0.05	-1.52	0.13	
Response Type 1 : Predictability (Low)	1.4	0.2	6.93	< .001	***
Response Type 2 : Predictability (Low)	-1.35	0.53	-2.56	< .05	*
Noise 1 : Predictability (Low)	0.79	0.34	2.32	< .05	*
Noise 2 : Predictability (Low)	0.47	0.25	1.88	0.06	.
Response Type 1 : Noise 1	0.79	0.16	4.88	< .001	***
Response Type 2 : Noise 1	-0.61	0.42	-1.44	0.15	
Response Type 1 : Noise 2	-0.15	0.15	-0.95	0.34	
Response Type 2 : Noise 2	-0.28	0.33	-0.83	0.41	

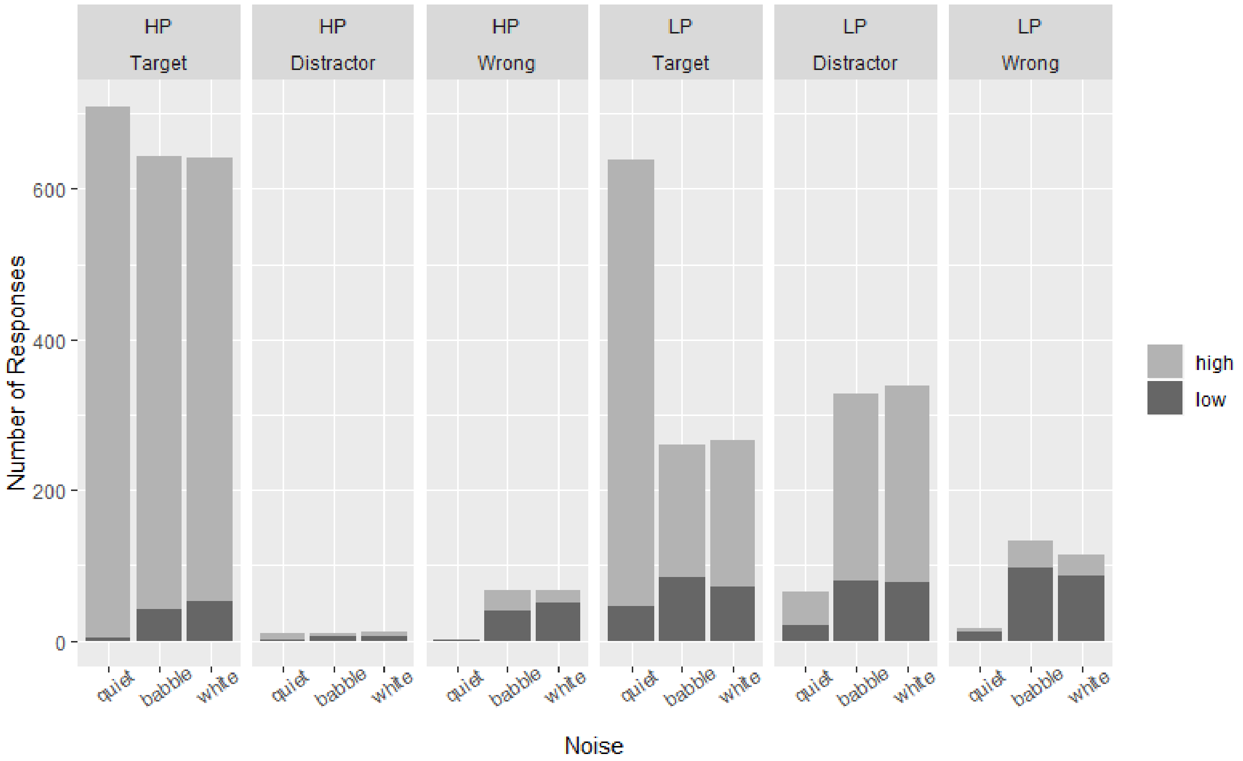

Figure 1. *For all answer types (Target, Distractor, and Wrong) and predictability levels (High Predictability HP and Low Predictability LP) the responses are presented per noise type (Quiet, Babble, and White), showing high and low confidence ratings.*

References

Bates, D., Maechler, M., Bolker, B., & Walker, S. (2015). Fitting linear mixed-effects models using lme4. *Journal of Statistical Software*, *67*(1), 1–48. doi:10.18637/jss.v067.i01

Failes, E., Sommers, M. S., & Jacoby, L. L. (2020). Blurring past and present: Using false memory to better understand false hearing in young and older adults. *Memory & Cognition, 48*(8), 1403-1416. doi: 10.3758/s13421-020-01068-8

R Development Core Team (2022). *R: A Language and Environment for Statistical Computing.* Vienna, Austria: R Foundation for Statistical Computing.

Rogers, C. S., Jacoby, L. L., & Sommers, M. S. (2012). Frequent false hearing by older adults: the role of age differences in metacognition. *Psychology and Aging, 27*(1), 33. doi: 10.1037/a0026231

Sommers, M. S., Morton, J., and Rogers, C. (2015). "You are not listening to what I said: false hearing in young and older adults," in *Remembering: Attributions, Processes, and Control in*

Human Memory (Essays in Honor of Larry Jacoby). Eds. D. S. Lindsay, C. M. Kelley, A. P. Yonelinas and H. L. Roediger III. New York, NY: Psychology Press, 269-284).

Van Os, M., Kray, J., & Demberg, V. (2021). Mishearing as a Side Effect of Rational Language Comprehension in Noise. *Frontiers in Psychology, 12,* 1-17. doi: 10.3389/fpsyg.2021.679278
